# Developmental differences in the perception of naturalistic human movements

**DOI:** 10.3389/fnhum.2022.1046277

**Published:** 2023-01-10

**Authors:** Ioannis Ntoumanis, Anna Shestakova, Maria Koriakina, Dzerassa Kadieva, Grigory Kopytin, Iiro P. Jääskeläinen

**Affiliations:** ^1^International Laboratory of Social Neurobiology, Institute for Cognitive Neuroscience, HSE University, Moscow, Russia; ^2^Federal State Budgetary Institution the Turner Scientific Research Institute for Children’s Orthopedics Under the Ministry of Health of the Russian Federation, Saint-Petersburg, Russia; ^3^Brain and Mind Laboratory, Department of Neuroscience and Biomedical Engineering, Aalto University School of Science, Espoo, Finland

**Keywords:** EEG, intersubject correlation, development, motion perception, naturalistic stimuli

## Abstract

**Introduction:**

It is widely believed that we are more attentive towards moving versus static stimuli. However, the neural correlates underlying the perception of human movements have not been extensively investigated in ecologically valid settings, nor has the developmental aspect of this phenomenon. Here, we set forth to investigate how human limb movements displayed in naturalistic videos influence the attentional engagement of children and young adults.

**Methods:**

Thirty-nine healthy participants (4–26 years old) were presented with naturalistic videos featuring human goal-directed movements, while neural activity was recorded using electroencephalography (EEG). Video scenes were automatically annotated as containing arm, leg or no movement, using a machine learning algorithm. The viewers’ attentional engagement was quantified by the intersubject correlation of EEG responses evoked by the videos.

**Results:**

Our results demonstrate that scenes featuring limb movements, especially simultaneous arm and leg movements, elicit higher attentional engagement than scenes with no limb movement. Interestingly, this effect was found to diminish with age.

**Discussion:**

Overall, our findings extend previous work on the perception of human motion by implementing naturalistic stimuli in the experimental design and extend the list of factors influencing the viewer’s engagement exerted by naturalistic videos.

## 1. Introduction

For more than a century, moving stimuli have been believed to attract our attention more than static stimuli (Pillsbury, 1908; Washburn and Putney, 1998). Stimulus movements “force attention on us; they take us by storm, and we can offer no resistance” (Titchener, 1915). This phenomenon has played a crucial role for the development of species, since one’s ability to detect biological movements in one’s surroundings can be a matter of life or death in such extreme situations as that of being close to a lurking predator in the jungle. Earlier research on biological motion perception has relied on point-light displays of biological motion (e.g., Carter and Pelphrey, 2006), which differ markedly from real-world movements, leaving our understanding of how the perception of human motion develops incomplete. Here, we investigate the perception of human limb movements displayed in naturalistic videos, while accounting for the developmental aspect of this phenomenon.

The perception of biological motion is often considered an automatic function due to its significance for survival (Thompson and Parasuraman, 2012). For instance, despite their limited visual attention skills, newborns are able to discriminate between point-light displays of biological versus non-biological motion (Simion et al., 2008). However, there are factors that appear to influence the perception of human movements, such as attention, motor, and visual experience (Jacobs et al., 2004; Thompson and Parasuraman, 2012). A characteristic of human actions is that they are defined by certain features, such as arms and legs that move in a specific way (Thompson and Parasuraman, 2012). Tracking these features requires both bottom-up (e.g., detect motion based on luminance modulation) and top-down processing (e.g., attentional tracking; Lu and Sperling, 1995; Whitney, 2006).

Importantly, both bottom-up and top-down processing have been found to improve with age, partly due to the maturation of ventral and dorsal visual streams (Parrish et al., 2005; Ghanouni et al., 2015). This is believed to lie beneath the development of motion perception with aging (Allison et al., 2000; Blakemore, 2012; Ghanouni et al., 2015). There are additional functional changes that occur in the brain during development that may affect biological motion perception. For example, the superior temporal sulcus (STS), which is a brain region activated during the perception of biological motion (Allison et al., 2000; Grossman et al., 2000; Grezes et al., 2001; Frith, 2007; Blakemore, 2012), is considered to undergo protracted development during adolescence (Blakemore, 2012). The specificity of STS for biological motion perception has thereby been found to increase with age (Carter and Pelphrey, 2006). In general, adults are more sensitive to biological motion than young children (Bertenthal and Pinto, 1993; Pinto, 2006; Blake and Shiffrar, 2007). However, given that the above conclusions have been largely driven by studies implementing point-light displays of biological motion, it is important to further investigate the developmental aspect of the perception of human movements in an ecologically valid setting.

Naturalistic videos mimic the real world while being diverse and dynamic (Nastase et al., 2019). On the one hand, such stimuli are considered interesting and engaging (Lehne et al., 2015; Bezdek et al., 2017) and on the other hand, they allow us to decipher the neural dynamics in close-to-real-life settings (Saarimäki, 2021). Neuroimaging data corresponding to naturalistic stimuli are usually not analyzed with explicit response models, since it is challenging to build predictors of specific events (Nastase et al., 2019; Jääskeläinen et al., 2021). Instead, model-free approaches are preferred, such as intersubject correlation (ISC) analysis (Hasson et al., 2004; Dmochowski et al., 2012). Earlier research suggests that electroencephalography (EEG) ISC covaries with the attentional state of the subjects, with attentionally engaging videos increasing the ISC of EEG responses (Dmochowski et al., 2012; Cohen and Parra, 2016; Ki et al., 2016). Interestingly, younger individuals have been found to exhibit higher ISC (Petroni et al., 2018).

In the present study, we used a machine-learning algorithm to detect video scenes featuring arm or leg human movement, and we further assessed how these features influence the ISC of EEG responses to the video stimuli. Given that moving stimuli have been associated with increased attention, we hypothesized that video scenes displaying limb movements elicit higher ISC than scenes displaying no limb movement. Furthermore, since sensitivity to biological motion has been found to increase with age, we hypothesized that ISC is susceptible to video limb movements in a pronounced way in older participants.

## 2. Materials and methods

### 2.1. Participants

Twenty-three healthy children (11 females, aged 4–16 years, mean age = 10.34) and 16 healthy adults (10 females, aged 18–26 years, mean age = 20.19) participated in the experiment. The under-aged participants were accompanied by their parents. Informed consent was obtained from all participants or their legal guardians. The study was approved by the Institutional Review Board of the local ethics committee. Overall, the experiment was carried out in accordance with the recommendations of the Declaration of Helsinki and its amendments.

### 2.2. Stimuli

Each participant was presented with 83 silent clips integrated into 4 video blocks of 4 min each. The order of the blocks was randomized across participants, whereas the order of the clips within each block was fixed (clips were integrated in a single.mp4 file presented to the subjects). The mean duration of the clips was 16 s and there was no narrative structure across them. There was no gap between consecutive clips of the same video block, and participants received no instruction about whether they should re-center their gaze before each clip starts. The background color of the videos was black. Each video included scenes with human motor activity, such as a child engaged in sports, as well as scenes without motor activity (this condition included both scenes featuring immobile humans and scenes with no human content, such as universe footage; Supplementary Table 1). The video stimuli can be found in Supplementary Videos 1–4.

Notably, the videos were presented in a silent mode, because the participants were simultaneously presented with a non-attended auditory oddball stimuli, the results of which will be separately reported elsewhere. Although performing distracting tasks while watching videos has been found to diminish neural synchronization of the subjects (Cohen et al., 2018), the motor-related visual information could be retrieved by the participants even under the potential distraction caused by the oddball task. Notably, the oddball task was not consistent across videos or participants, eliminating the possibility that the oddball task has confounded the data presented in this article.

### 2.3. EEG data collection and preprocessing

Electroencephalography activity was recorded by means of 32 electrodes at a sampling frequency of 500 Hz for children. The adults’ brain activity was recorded by simultaneous EEG and MEG recording, yet in the current study we only report the EEG data which contained the signals of 64 electrodes at a sampling frequency of 1,000 Hz (which was downsampled at 500 Hz). In order to not bias the ISC analysis, we analyzed only the recordings of the 32 electrodes the two groups had in common. Moreover, because scene transitions in the videos could causes increases in the ISC that are not related to stimulus movements, we removed the EEG data corresponding to the first 5 s of each short clip within each video block.

The EEG preprocessing pipeline followed previous studies (Dmochowski et al., 2012; Cohen and Parra, 2016). First, the EEG segments corresponding to the duration of each video block were extracted and temporally aligned across subjects. Then, the signals were high-pass filtered at 1 Hz and low-pass filtered at 50 Hz. Next, the channels whose average power exceeded the mean channel power by 4 SDs were identified and replaced with zero valued samples, so that these channels do not affect the calculation of the covariance matrices. Eye-movement related artifacts were removed by Independent Component Analysis (ICA), using the fastICA algorithm (Hyvärinen, 1999). Outliers were replaced with zero, as well as the samples 40 ms around them (before and after). As outliers we classified the samples whose magnitude exceeded 3 SDs of the mean magnitude of their corresponding channel. Lastly, the time course of each channel and each subject was *z*-scored. Provided that children and adults were recorded with different EEG systems, we *z*-scored the time courses in order to control for any between-groups confounding factors. This step is typical in fMRI ISC studies (Nastase et al., 2019).

### 2.4. Intersubject correlation analysis

The intersubject correlation was measured *via* a correlated component analysis. Parra et al. (2018) offer a detailed description of the method. First, the data from all participants were concatenated for each video. Based on the concatenated data, between-subject and within-subject covariance matrices were computed for each stimulus. These matrices were then averaged over the four video blocks, so that all stimuli correspond to the same projection vectors (Cohen and Parra, 2016). After the optimization of the correlated components, we calculated the ISC of each subject in a leave-one-out approach. This resulted in a single number per participant, reflecting the level at which this participant’s neural activity was synchronized with the neural activity of all other participants. The reported ISC values correspond to the sum of the three most correlated components, following previous studies (Cohen and Parra, 2016; Iotzov et al., 2017; Cohen et al., 2018; Petroni et al., 2018), allowing us to measure the overall level of neural synchronization regardless of each component’s anatomical origin. We also computed the ISC over sliding time windows, in order to assess the dynamics of ISC as a function of the motor-related content of short video scenes. To that end, the recordings were divided into 1.5 s sliding windows, with 1.2 s overlap (300 ms resolutuion). The ISC of each time window was then calculated based on the previously estimated projection vectors W (Dmochowski et al., 2012). Code for conducting correlated component analysis has been previously published by Parra Lab (https://www.parralab.org/isc/).

Previous EEG ISC studies calculated the time-resolved ISC in time windows of 5 s (e.g., Dmochowski et al., 2012; Poulsen et al., 2017), while a recent analysis showed that ISC can be reliably measured on a time-scale of 10 s (Madsen and Parra, 2022). However, multiple naturalistic movements are possible to be displayed consecutively within such a long period, making it difficult to determine which type of movement was dominant within each time window. On the contrary, 1.5 s is a reasonable period for capturing individual quick movements of the videos’ actors (based on our data, the average duration of arm movements was 1.36 s and the average duration of leg movements was 9.20 s), while achieving a reliable neural synchrony estimation (here, 1.5 s correspond to 750 time samples).

### 2.5. Automatic annotation of movements

The detection of arm and leg movements in the video stimuli was achieved in two steps. First, a machine learning algorithm was employed to detect the arms and legs in each video frame of our stimuli (OpenPose demo; Cao et al., 2018). This algorithm is robust to multiple scales and to multiple people displayed on screen. Second, based on the Euclidean distance of each limb’s screen coordinates between consecutive video frames, we detected the frames containing arm or leg movement. The Euclidean distance had to be between two thresholds, so that we interpret that a movement occurred. The lower threshold served to filter out small, non-significant movement, like when the camera was not totally stable. The upper threshold served to filter out scene transitions. The optimal thresholds were selected by visually inspecting the output, since there is no ground truth in terms of when a movement occurred. This procedure resulted in a time series of motion indicators, which was then temporally aligned with the time-resolved ISC. That is to say, we determined during which time windows an arm or a leg movement was displayed. This analysis also excluded the first 5 s of each short clip within each video block, so that it is aligned with the ISC analysis. In general, employing a machine learning algorithm to track movements of human limbs has been found to be promising in studies of naturalistic human movements (Peterson et al., 2021). For the automatic annotation of movements, we implemented the OpenPose github repository (https://github.com/CMU-Perceptual-Computing-Lab/openpose), as in Ntoumanis et al. (2022).

### 2.6. Average luminance difference

A comparison of the ISC between scenes with human movements and scenes without human movements might be confounded by the fact that the former may contain higher level of visual dynamics, in general, compared to the latter. Therefore, we quantified the visual dynamics of the videos, using the Average luminance difference (ALD) across time, in order to examine the effect of human movements on ISC, after controlling for the overall visual dynamics. The ALD was calculated as in Poulsen et al. (2017). Specifically, the four videos were first converted to gray scale (0–255) by averaging over the RGB color channels. Then, we calculated the squared difference in pixel intensity between consecutive frames and calculated the average across pixels. The obtained ALD time series was then downsampled to match the temporal resolution of the ISC. The downsampling was done based on the maximum ALD per time window (Poulsen et al., 2017). This analysis also excluded the first 5 s of each short clip within each video block, so that it is aligned with the ISC analysis. Supplementary Figure 1 illustrates the correlation between ALD and ISC per component. Supplementary Figure 2 shows that there was no difference in ALD between scenes with different motor-related content.

### 2.7. Statistical analysis

**FIGURE 1:**
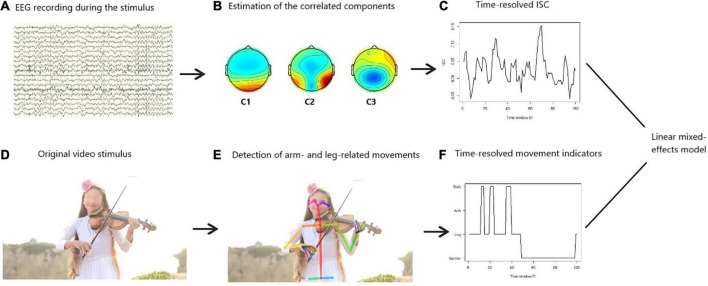
Schematic representation of the data analysis. Based on the preprocessed electroencephalography (EEG) signals **(A)**, we extracted components that are maximally correlated among participants **(B)**. Using these components, we projected the EEG data to the so-called components’ space and measured the intersubject correlation (ISC). We did this in a time-resolved fashion, i.e., repetitively for sliding time windows of 1.5 s size and 80% overlap. This revealed how the ISC changed across time **(C)**. Furthermore, we applied a machine-learning algorithm to the original video stimulus **(D)** in order to detect scenes in the videos where arm- or leg-related movements occur **(E)**. We did this in a time-resolved fashion, i.e., repetitively for sliding time windows of 1.5 s size and 80% overlap **(F)**. Therefore, the time series displayed on panels **(C,F)** were perfectly temporarily aligned. Then, we estimated a linear mixed-effects model to predict the ISC based on the time-resolved movement indicators, while participants’ age was included as a covariate.

After obtaining the time-resolved ISC and the time-resolved motion indicators, we estimated a linear mixed-effects model, with subject-level random effects (Bates et al., 2015), in order to predict ISC. Scenes with neither arm nor leg movement served as the reference level of the motor-related content of the stimulus and the occurrence of arm or leg (or both) movements were included as regressors. The model also assessed whether or not participants’ age moderates the relationship between ISC and human movements displayed in the naturalistic videos. Finally, the ALD was also included in the model as a covariate, so that the effect of naturalistic movements on ISC is examined without the confound of general visual dynamics. A mixed-effects model was preferred over a typical fixed-effects one, because ISC has been found to significantly vary across individuals (Iotzov et al., 2017). Figure 1 illustrates the overall data analysis procedure.

## 3. Results

First, we estimated the components of the EEG signals that capture maximally correlated responses across subjects (Figure 1B). These component topographies were found similar to previous studies (Dmochowski et al., 2012, 2014; Cohen and Parra, 2016; Iotzov et al., 2017; Cohen et al., 2018; Petroni et al., 2018). For instance, the first two components revealed a strong positivity at occipital sites, which is consistent with visual processing (Figure 1B). This suggests that the highest ISC during video watching was achieved by similar processing of the visual stimuli. Overall, based on the scalp topographies the estimated correlated components were moderately perceptual and cognitive and not predominantly motor (Figure 1B).

A linear mixed-effects model with subject-level random effects was estimated in order to predict ISC based on the displayed human movements and participants’ age, while accounting for visual dynamics. The results are summarized in Table 1. First of all, children exhibited significantly higher ISC compared to adults (*p* = 0.009), consistently with previous studies (Petroni et al., 2018). Also, the ISC was found to significantly increase with ALD (*p* < 0.0001), which is a measure of visual dynamics. This finding is also in line with earlier research (Poulsen et al., 2017). Moreover, displayed arm movements and leg movements were found to increase the ISC (*p* < 0.0001 and *p* = 0.003, respectively), especially when presented simultaneously (*p* < 0.0001). Importantly, participants’ age significantly moderated the effect of simultaneous arm and leg movements on ISC (*p* < 0.0001).

**TABLE 1:** Linear mixed effects model for predicting the intersubject correlation (ISC) with subject-level random effects.

Effect	Est.	S.E.	*t*-value	d.f.	*p*
(Intercept)	0.004	0.001	3.942	46.406	<0.0001
Children	0.004	0.001	2.720	43.945	0.009
Arm	0.003	0.001	4.491	83,778.017	<0.0001
Leg	0.002	0.001	2.924	83,782.516	0.003
Both	0.004	0.001	6.309	83,778.033	<0.0001
ALD	0.002	0.000	8.650	83,779.217	<0.0001
Children × Arm	0.001	0.001	0.893	83,778.786	0.372
Children × Leg	0.003	0.001	3.911	83,787.926	<0.0001
Children × Both	0.007	0.001	7.686	83,779.647	<0.0001

Data from all the four video stimuli were included in the analysis. The reference level of the variable movement was “neither” (i.e., scenes featuring neither arm nor leg movements). The reference age group was adults.

**FIGURE 2:**
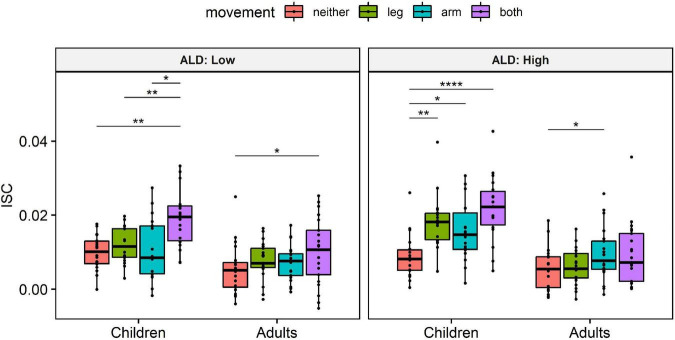
Average intersubject correlation (ISC) by movement, Age group and Average luminance difference (ALD) levels. Data from all 4 video stimuli were included in the analysis. Due to the problem of *p*-values associated with large samples (Lin et al., 2013), we first averaged the data of each participant and only then applied pairwise one-sample Wilcoxon tests, in order to limit the probability of Type I error. Wilcoxon tests were preferred over *t*-tests due to the non-normal distribution of the data (as assessed by Shapiro–Wilk test, *p* < 0.05). Hence, individual points in the plot correspond to individual subjects (sum of 3 strongest components). *P*-values were corrected for multiple comparisons, using Bonferroni correction. *p < 0.05, **p < 0.01, and ****p < 0.0001.

In order to better understand the significant interaction between age and motion indicators on ISC, while accounting for ALD, we conducted pairwise one-sample Wilcoxon tests. Specifically, we first averaged the data of each participant within each condition, in order to limit the probability of Type I error, which has been associated with large samples (Lin et al., 2013). Then, we transformed the continuous ALD to a binary variable, using the median ALD as a cut-off value (median ALD = 0.64). Then, for each level of ALD (Low/High) and Age group (Children/Adults), we compared the ISC between different movements (Figure 2). This *post hoc* analysis revealed that children exhibit significantly lower ISC during scenes with no movements compared to scenes with arm movements, scenes with leg movements and scenes with simultaneous arm and leg movements. Importantly, this effect was present both during scenes with high- and scenes with low-level of visual dynamics. On the other hand, adults’ ISC was found to be less sensitive to scenes featuring naturalistic human movements, as reflected in the corresponding pairwise comparisons (Figure 2).

## 4. Discussion

To our knowledge, this is the first time that developmental differences in the neural correlates underlying the perception of human movements are studied in an ecologically valid setting. Specifically, the aim of our work was to compare the level of attentional engagement between video scenes featuring different human limb movements, while taking into account participants’ age. To that end, we estimated the ISC of EEG responses to the stimuli, which is considered to be a marker of neural engagement and attention (Dmochowski et al., 2012; Cohen and Parra, 2016; Ki et al., 2016; Cohen et al., 2018).

There are three important findings of our study. First, in line with our hypothesis, displayed arm and leg human movements were found to increase attentional engagement. Thus, our data support earlier studies linking moving stimuli to enhanced attention (Pillsbury, 1908; Titchener, 1915; Washburn and Putney, 1998), and extend previous work on the perception of point-light displays of biological motion by implementing naturalistic stimuli in the experimental design.

Second, attentional engagement was found to decrease with participants’ age, regardless of the motor-related content of the stimuli. This finding supports previous studies showing that neural activity becomes more variable with maturity while passively watching real-world stimuli (Campbell et al., 2015; Petroni et al., 2018). Campbell et al. (2015) argue that the observed phenomenon might be regarded to different patterns of attention, especially reflected in the superior frontal lobe and intraparietal sulcus. However, the low spatial resolution of the EEG technique does not allow us to test this postulation. We speculate that older participants have developed the cognitive capacity to interpret the videos more idiosyncratically than young children, which is also reflected in a more idiosyncratic brain activity (i.e., lower ISC).

Third, we found that the effect of stimulus movements on viewers’ attentional engagement is moderated by participants’ age. In fact, ISC was found to be susceptible to simultaneous arm and leg movements in a pronounced way in children. This runs counter to our hypothesis of observing the opposite effect, which was based on the lower sensitivity to human motion that has been observed in young children (Bertenthal and Pinto, 1993; Carter and Pelphrey, 2006; Pinto, 2006; Blake and Shiffrar, 2007). While asking the participants to rate their interest in each condition would better address the role of interest in the perception of limb movements, a possible explanation of our finding is that scenes featuring simultaneous arm and leg movements were more interesting than scenes with no limb movement to young children, whereas for older participants all scenes were equally interesting. Essentially, ISC is not only a marker of attention, but also a marker of interest (Nastase et al., 2019). This speculation is supported by previous studies positing improved saccadic responses to interesting versus non-interesting stimuli for young but not older children (Irving et al., 2011).

Earlier fMRI studies suggest that age affects the biological motion perception, which is particularly reflected in the STS brain activity (Grossman et al., 2000; Grezes et al., 2001; Carter and Pelphrey, 2006; Ghanouni et al., 2015). In the present study, by using EEG ISC as a marker of attentional engagement, we reveal an additional role that age might play in the perception of human movements. That is to say, age might essentially influence one’s attentional capacities which in turn, influence the perception of human motion. This postulation is supported by earlier research showing that young children have reduced attention span (Koriakina et al., 2021) and are worse in allocating attention simultaneously to multiple targets (Dye and Bavelier, 2010).

From the evolutionary point of view, motion perception has been necessary for humans, not only for survival (Johansson, 1975), but also for social interactions (Brüne and Brüne-Cohrs, 2006). In fact, there is evidence that Theory of Mind, a higher-order social perception skill, evolved from the capacity to monitor biological motion (Premack and Woodruff, 1978). Hence, the higher ISC that we found during scenes with arm and leg movements compared to scenes with no movements could also be explained by the fact that limbs are often used in social communications. In other words, our evolutionary ability to understand other people’s intentions seems to be supported by our increased attention to body movements that facilitate communication. This is also supported by our finding that biological movements direct our attention even during scenes with low level of visual dynamics.

Finally, our results expand the list of factors influencing the ISC exerted by naturalistic videos. According to previous studies, the viewers’ engagement with videos depends on the emotional content, suspense and cognitive demand for processing complex dynamic information (Potter and Bolls, 2012; Sukalla et al., 2016; Jääskeläinen et al., 2021), as well as on the viewers’ personality traits (e.g., Bacha-Trams et al., 2018). Here, we demonstrate that engagement with naturalistic videos is also influenced by the presentation of human limb movements in the videos.

Certain limitations of this study need to be taken into account. For instance, the videos presented to the participants did not contain any narrative structure. An interesting story narrated in a movie would take the viewers on a journey and might evoke cognitive and emotional responses overweighting the ones evoked solely by the actors’ movements (Richardson et al., 2020). Future studies may annotate such videos to compare the level of attentional engagement between different motor-related categories of scenes. Notably, the scenes of the videos that contained no movements were mainly (but not only) nature scenes, with no clear objects/subjects to attract attention and smoother visual dynamics compared to scenes featuring human movements. However, given that the ALD (which is a measure of visual dynamics) was found to not be increased by the presentation of movements and it was also included as a covariate in our regression model, it is unlikely that this has confounded our findings. It is well known that experiments using naturalistic stimuli are not fully controlled, because of the complexity and richness of such stimuli (Jääskeläinen et al., 2021). Hence, we cannot exclude the possibility that there was some confound in scenes with movements, which was absent from scenes without movements, which might have affected the observed effects. However, by using a high variety of video clips (83 in total), we aimed to eliminate this possibility. In addition the machine learning algorithm that we used to annotate the videos allowed us to examine specifically the effect of arm and leg movements, instead of examining only the effect of movements, in general. Moreover, we conducted the ISC analysis in time windows of 1.5 s, although it has been found that longer time windows may capture a wider range of the ISC (Madsen and Parra, 2022). We did this in order to better fit the motor-related content of the video, at the cost of decreasing the reliability of the ISC. In addition, the order of the short clips within each video block was fixed, yet we addressed this issue by excluding the first 5 s of each short clip from our data analysis. Finally, it would be promising for future studies to investigate the generalizability of these results to a wider population, including more infants, but also older people (e.g., >50 years old).

Taken together, our results extend previous work on the perception of human motion by implementing naturalistic stimuli in the experimental design. Moreover, by examining the developmental aspect of the observed phenomena, we showed that attentional engagement is susceptible to naturalistic limb movements in a pronounced way in older participants. Our findings may be used to improve advertising and health-related video spots by implementing human movements to increase viewers’ attentional engagement.

## Data availability statement

The raw data supporting the conclusions of this article will be made available by the authors, without undue reservation.

## Ethics statement

The studies involving human participants were reviewed and approved by the Institutional Review Board, Higher School of Economics, Moscow, Russia. The patients/participants provided their written informed consent to participate in this study.

## Author contributions

IN, AS, and IJ designed research. IN, MK, DK, and GK performed research. IN analyzed the data and wrote the manuscript. All authors contributed to the article and approved the submitted version.
