# Supplementary material for: Developmental differences in the perception of naturalistic human movements
Source: Front Hum Neurosci. 2023 Jan 10;16:1046277. doi: 10.3389/fnhum.2022.1046277 (PMC9872020; doi:10.3389/fnhum.2022.1046277)
Supplement: Supplementary file 1 [file Data_Sheet_1.docx]

# *Supplementary Materials*

**
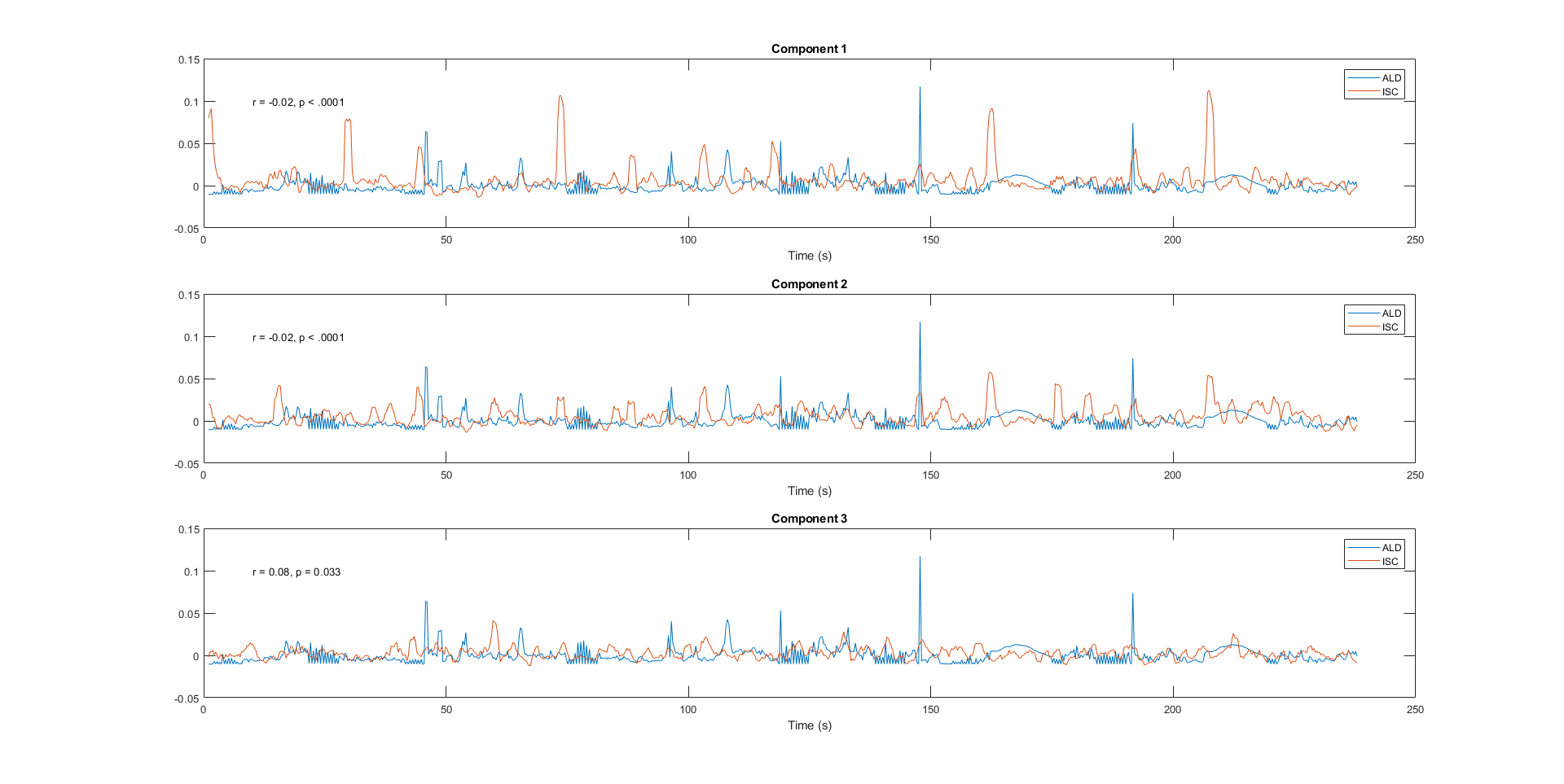
**

**Supplementary Figure 1.** Correlation between ALD and ISC per component. The localization of the first two correlated components was occipital (see Figure 1B), positing that they capture the ISC due to consistent visual processing of the visual stimulus. This explains why in this figure the correlation between ALD and the two strongest components is statistically significant. Overall, this results is in line with Poulsen et al. (2016).


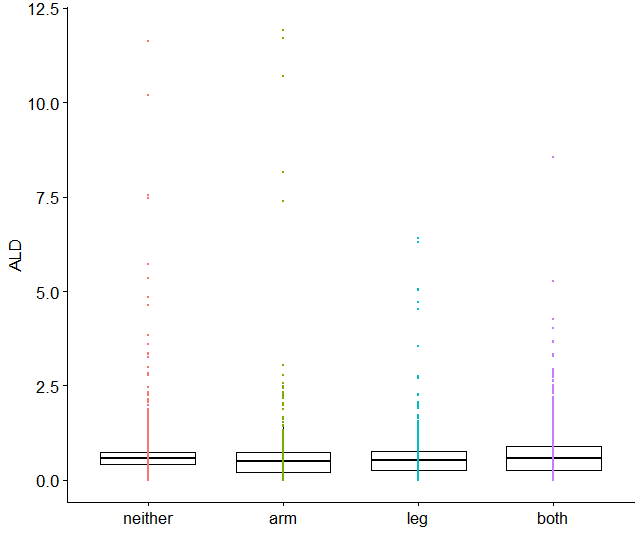


**Supplementary Figure 2.** Comparison of ALD between conditions. The average ALD values per condition are 0.61 for scenes featuring arm movements (Arm), 0.62 for scenes featuring leg movements (Leg), 0.73 for scenes featuring both arm and leg movements (Both) and 0.67 for scenes featuring neither arm nor leg movements (Neither). Due to the highly unbalanced sample sizes, we avoided conducting a statistical test to compare the ALD between conditions. However, the average values in combination with the boxplot provide evidence that there was no significant difference in ALD between the conditions.

**Supplementary Table 1**

*Proportion of arm movement, leg movement, simultaneous arm and leg movement (both) and neither arm nor leg movement (neither), shown in each video. Notably, these four sets of recording samples are disjoint.*

|  | Movement | | | |
| --- | --- | --- | --- | --- |
|  | arm | leg | both | neither |
| video 1 | 14% | 16% | 28% | 42% |
| video 2 | 23% | 26% | 12% | 39% |
| video 3 | 22% | 7% | 25% | 46% |
| video 4 | 14% | 37% | 17% | 32% |
| Total | 18.25% | 21.50% | 20.50% | 39.75% |
